# The Apolipoprotein C-I Content of Very-Low-Density Lipoproteins Is Associated with Fasting Triglycerides, Postprandial Lipemia, and Carotid Atherosclerosis

**DOI:** 10.1155/2011/271062

**Published:** 2011-07-06

**Authors:** John-Bjarne Hansen, José A. Fernández, Ann-Trude With Notø, Hiroshi Deguchi, Johan Björkegren, Ellisiv B. Mathiesen

**Affiliations:** ^1^Hematology Research Group, Department of Clinical Medicine, University of Tromsø, 9037 Tromsø, Norway; ^2^Department of Molecular and Experimental Medicine, The Scripps Research Institute, La Jolla, CA 92037, USA; ^3^The Computational Medicine group, Atherosclerosis Research Unit, Department of Medicine, Karolinska Institute, Stockholm, Sweden; ^4^Cerebrovascular Diseases and Atherosclerosis Research Group, Department of Clinical Medicine, University of Tromsø, Tromsø, Norway

## Abstract

*Background*. Experimental studies in animals suggest that apolipoprotein (apo) C-I is an important regulator of triglycerides in fasting and postprandial conditions and associated with carotid atherosclerosis. *Methods*. A cross-sectional study was conducted with 81 subjects, aged 56–80 years recruited from a population health survey. The participants underwent a fat tolerance test (1 g fat per Kg body weight) and carotid atherosclerosis was determined by ultrasound examination. VLDL particles, Sf 20–400, were isolated and their lipid composition and apoC-I content determined. *Results*. The carotid plaque area increased linearly with the number of apoC-I molecules per VLDL particles (*P* = 0.048) under fasting conditions. Fasting triglycerides increased across tertiles of apoC-I per VLDL particle in analyses adjusted for apoC-II and -C-III, apoE genotype and traditional cardiovascular risk factors (*P* = 0.011). The relation between apoC-I in VLDL and serum triglycerides was conveyed by triglyceride enrichment of VLDL particles (*P* for trend <0.001. The amount of apoC-I molecules per VLDL was correlated with the total (*r* = 0.41, *P* < 0.0001) and incremental (*r* = 0.35, *P* < 0.001) area under the postprandial triglyceride curve. *Conclusions*. Our findings support the concept that the content of apoC-I per VLDL particle is an important regulator of triglyceride metabolism in the fasting and postprandial state and associated with carotid athrosclerosis.

## 1. Introduction

Growing evidence, based on genetically engineered mice models and a polymorphism in the promoter region of apoC-I in humans (HpaI) associated with increased expression of apoC-I transcription, supports the concept that apoC-I plays a pivotal role for regulation of triglycerides in fasting and postprandial conditions. The presence of the apoC-I gene is shown to increase fasting and postprandial triglycerides compared to apoC-deficient mice independent of the apoE gene status [1] and the apoC-III gene status [2]. Likewise, overexpression of the human apoC-I gene in mice (*APOC1*) promotes elevated triglyceride levels, mostly attributed to increased levels of very-low-density lipoproteins (VLDLs) [3, 4]. Furthermore, the presence of the *HpaI* polymorphism in humans is associated with increased triglyceride levels [5, 6]. Experimental studies have shown that ApoC-I modulates lipid metabolism by increasing the production rate of hepatic VLDLs [1], inhibition of lipoprotein lipase activity [1, 7, 8], interference with the apoE-mediated uptake of VLDLs [5, 9], and inhibition of cholesteryl ester transfer protein (CETP) [10, 11]. 

ApoC-I is primarily expressed in the liver [12] and secreted into plasma as a 6.6 kDa protein where 60–70% is associated with high-density lipoprotein (HDL) and 30–40% associated with VLDL under fasting conditions [13]. Total plasma levels of apoC-I are increased in patients with hypertriglyceridemia [13, 14] and decreased in patients with Tangier's disease [15]. During postprandial elevation of triglyceride-rich lipoproteins (TRLs), apoC-I is transferred from HDL to VLDL (Svedberg flotation (Sf) 20–400) [16] without affecting total plasma levels of apoC-I [17].

Data from clinical studies suggest that the content of apoC-I molecules per VLDL particle in the fasting [18] and postprandial state [19, 20] is a novel risk factor for atherosclerosis and coronary artery diseases (CAD). VLDL particles are enriched with apoC-I in patients with CAD [19], in healthy individuals with increased intima-media thickness (IMT) [21] and are an independent predictor for IMT [20] and the size of carotid atherosclerotic plaques [18]. However, the impact of phenotypic expression of apoC-I in VLDLs on lipid metabolism under fasting and postprandial conditions is not known. To address this question, we determined the content of apoC-I per VLDL molecule and related them to serum lipids and LPL activity in the fasting and postprandial conditions among subjects with and without carotid atherosclerosis recruited from a general population.

## 2. Materials and Methods

### 2.1. Study Participants

The participants of the study were recruited from a population health study (the fifth survey of the Tromsø study in 2001), which included ultrasound examination of the right carotid artery. Persons aged 56–80 years old were eligible for the plaque group of the present study if they had at least one plaque with a thickness of ≥2.5 mm in the carotid bifurcation or internal carotid artery, and for the plaque-free group if they had no plaques in the carotid arteries. Persons who responded positive to our invitation letter were invited to a screening visit. At the screening visit, a complete medical history, physical examination, and blood samples were taken with special emphasis on exclusion criteria. Exclusion criteria were any of the following conditions; regular use of lipid-lowering drugs (HMG-CoA reductase inhibitors, resins, or nicotinic acid derivates) or oral anticoagulants, cancer, or other serious life-threatening medical conditions, hypothyroidism, renal, hepatic, or psychiatric disease, and current abuse of alcohol or drugs. 

A detailed interview on the occurrence of cerebrovascular and cardiovascular events, defined as prior or present transient ischemic attacks (TIAs), stroke, amaurosis fugax, angina pectoris, myocardial infarction, peripheral vascular disease, and diabetes, and smoking habits and drugs was obtained. Hypertension was defined as usage of antihypertensive medication or systolic pressure above 160 mmHg and/or diastolic pressure above 95 mmHg. Diabetes was self-reported or defined as fasting plasma glucose ≥7.0 mmol/L or non-fasting ≥11.1 mmol/L at two occasions. Height and weight were measured with the participants in light clothing without shoes; body mass index (BMI) was calculated as weight per height squared (kg/m^2^). Blood pressure was recorded in seated position by the use of an automatic device (Dinamap Vital Signs Monitor). Three recordings were made at 1-minute intervals, and the mean of the last two values is used in this report. Eligible persons were invited to a second visit, where ultrasound examination of both carotid arteries was performed, and the participants were subjected to a fat tolerance test. Eight of the eligible subjects had diabetes mellitus type II. None of these had medical treatment, but received advice on dietary restrictions only. Informed written consent was obtained from the participants, and the regional committee for medical research ethics approved the study. The study was performed at the Clinical Research Unit at the University Hospital of North Norway.

### 2.2. Ultrasound Examination

At the population health screening, high-resolution B-mode and color Doppler/pulsed-wave Doppler ultrasonography of the right carotid artery was performed as described previously [22, 23]. Assessment of plaque size and morphology was made in all plaques present in the near and far walls of the common carotid, the bifurcation, and the internal carotid arteries on both sides (12 locations). All examinations and measurements of all plaques were recorded on videotapes. Stored B-mode images were subsequently transferred to a personal computer and digitized into frames of 768 × 576 pixels of 256 grey levels each (0 = black and 256 = white) with the use of a commercially available video grabber card (meteor II/Matrox Intellicam). Measurements of plaque area were made with the use of the Adobe Photoshop image-processing program (version 7.0.1), by tracing the perimeter of each plaque.

### 2.3. Fat Tolerance Test

A fat-tolerance test was conducted using a test meal prepared from standard porridge cream containing 70% of calories from fat of which 66% saturated fat, 32% monounsaturated fat, and 2% polyunsaturated fat. The test meals were served with two teaspoons of sugar, cinnamon, and two glasses (150 mL each) of sugar-free juice. The test meals were freshly prepared each morning. A weight-adjusted meal (1 gram fat per kg body weight) was served at 8:00 a.m. and consumed over a 15-min period. The participants were allowed to drink 350 mL calorie-free beverages and eat an apple during the following 8 hrs.

### 2.4. Blood Collection, Isolation of Triglyceride-Rich Lipoproteins and Storage

Blood was drawn from an antecubital vein in the morning at 7:45 a.m, after 12 hours overnight fasting and 48 hrs refrain of exhaustive physical exercise and alcohol consumption, and then, 2, 4, 6, and 8 hours after the meal, using a 19-gauge needle in a vacutainer system with minimal stasis. Serum was prepared by clotting whole blood in a glass tube at room temperature for 1 hour and then centrifuged at 2000 g for 15 minutes at 22°C. Aliquots of 1 mL were transferred into sterile cryovials (Greiner labortechnik, Nürtringen, Germany), flushed with nitrogen, and frozen at −70°C until further analysis. 

VLDL was isolated by preparative ultracentrifugation in the fasting state and four hrs after ingestion of the standard high-fat meal (postprandial state). First, chylomicrons (CM) were removed from plasma by over layering 8 mL EDTA plasma with 5 mL of NaCl solution (density 1.006 kg/L NaCl solution with 0.02% sodium azide and 0.01% EDTA) in a cellulose nitrate tube (Beckman Instruments Inc., Calif, USA) and centrifuged in a Beckman SW40Ti swinging-bucket rotor at 20.000 rpm for 1 hr at 4°C. The CM, with Sf rates >400, was removed by aspiration. Second, plasma samples were relayered with 5 mL 1.006 kg/L NaCl solution and subjected to centrifugation at 40.000 rpm for 20 hrs at 20°C. VLDL, with Sf 20–400, was carefully removed by aspiration from the top of the tube. ApoB-48 was measured in random samples and revealed that the VLDL fractions contained substantially less than 10% apoB-48 mass compared to total apoB mass in both the fasting and postprandial states, implying that 5–15% of apoC-I within the VLDL fraction can be due to remaining chylomicron remnants [21]. VLDL fractions were divided into three aliquots in cryovials, flushed with nitrogen, and frozen at −70°C until further analysis.

### 2.5. Serum Lipids and Apolipoproteins Measurements

Serum lipids were analyzed on a Cobas Mira S (Roche Diagnostics, F. Hoffmann-La Roche Ltd., Basel, Switzerland) with reagents from ABX Diagnostics (Montpellier, France). Total cholesterol (CHOD-PAP) and triglycerides (GPO-PAP) were measured with enzymatic colorimetric methods. Low-density lipoprotein (LDL) and high-density lipoprotein cholesterol were measured by selective inhibition colorimetric assays (LDL cholesterol direct and HDL cholesterol direct, respectively, ABX Diagnostics). Serum apolipoprotein A-I (apoA-I) and apolipoprotein B (apoB) were analyzed by turbidimetry on Cobas Mira S with reagents from ABX Diagnostics. Serum apolipoprotein E (apoE) was measured by an enzyme-linked immunosorbent assay, Apo-Tek ApoE (PerImmune Inc., Rockville, Md, USA), and genotyping of apoE was performed according to Hixson and Vernier [24] with slight modifications. An enzymatic immunoassay was used to measure apoC-I in isolated VLDL fractions [25]. Apolipoprotein CII in serum was analyzed by turbidimetry with reagents from Kamiy Biomed Comp (Seattle, WA, USA). ApoC-I in serum (AssayMax Human Apolipoprotein C-I ELISA kit), and apoC-III (AssayMax Human Apolipoprotein C-III ELISA kit) in serum, and isolated VLDL fractions were measured by commercial available immunoassays according to the manufacturer's descriptions (Assaypro LLC, St. Charles, MO, USA).

### 2.6. Lipoprotein Lipase Measurements

Eight hours after ingestion of the test meal, blood was drawn into vacutainers (Becton Dickinson, Meylan, Cedex, France) containing heparin as anticoagulant, and the heparinized blood was immediately placed on ice. Unfractionated heparin was given as a bolus injection (100 IU/kg bodyweight) on the contralateral arm to mobilize LPL from the endothelial surface into the circulation. A second blood sample was obtained exactly 15 minutes after heparin administration and immediately placed on ice. Heparinized plasma was recovered within 30 minutes by centrifugation (2000 ×g for 10 min) at 4°C, divided into aliquots of 1.0 mL in cryovials, flushed with nitrogen, and frozen at −70°C until further analysis. LPL activity was determined as previously described [26, 27]. In short, 185 *μ*L sonicated emulsion of ^3^H-oleic acid-labelled triolein in 10% Intralipid (Fresenius Kabi) was used as substrate and incubated with 15 *μ*L heparinized plasma samples. Plasma samples were preincubated for 2 hrs on ice with 0.5 vol goat antibodies to hepatic lipase (HL) to suppress HL activity. LPL activity was expressed as mU/mL corresponding to nmol of fatty acid released per minute at 25°C [28]. LPL mass was measured in postheparin plasma with a commercial ELISA kit (MARKIT-M LPL ELISA, Dainippon Sumitomo Pharma Co., Ltd., Osaka, Japan) according to manufacturer's instructions. Preheparin plasma samples had the following values for the total study population: LPL activity 0.97 ± 0.37 mU/mL, LPL mass 83.6 ± 36.9 ng/mL, and specific activity 0.02 ± 0.02 mU/ng. Postheparin plasma samples had the following values for the total study population: LPL activity 138.4 ± 43.1 mU/mL, LPL mass 729.1 ± 293.7 ng/mL, and specific activity 0.20 ± 0.06 mU/ng.

### 2.7. Calculations and Statistics

The number of apoC-I per VLDL particle and apoC-III per VLDL particle were calculated by dividing apolipoprotein concentrations in density fraction Sf 20–400 by their respective molecular mass (apoB-100 = 549 kD, apoC-I = 6.6 kD, and apoC-III = 8.8 kD). The fraction molarity of apoC-I/apoC-III was then divided by the corresponding molarity of apoB. Continuous variables were tested for normal distribution, and logarithmically transformed in statistical analyses if not normally distributed. Differences in continuous variables from fasting to postprandial conditions were analyzed by paired *t*-test. The number of apoC-I particles per VLDL was divided in tertiles, and significance of linear trends across tertiles was tested by linear regression. Pearson's correlation coefficients were used to examine correlations between continuous variables. All analyses were performed using SPSS (SPSS Inc., Chicago, Ill, USA) for windows software, version 16.0. Two-sided *P* values (<0.05) were considered statistically significant. Results are expressed as means ± 1 SD unless otherwise stated. 

## 3. Results

Plasma VLDL particles, Sf 20–400, were isolated from all study participants by preparative ultracentrifugation before (fasting condition) and 4 hrs after (postprandial condition) intake of a standard high-fat meal and analyzed for triglycerides, total cholesterol, and apolipoproteins B, C-I, and C-III. 

Characteristics, traditional cardiovascular risk factors, the presence of carotid plaques, diabetes mellitus and cardiovascular diseases, and regular use of major cardiovascular drugs across tertiles of the number of apoC-I per VLDL particle under fasting conditions are shown in Table 1. Fasting triglycerides increased significantly across tertiles of apoC-I per VLDL particle in unadjusted analysis (*P* = 0.01) and after adjustment for age, gender, BMI, apoC-II, apoC-III, and apoE genotype (*P* = 0.012). Further adjustments for cardiovascular risk factors such as smoking, blood pressure, and serum lipids and apolipoproteins did not affect the relationship. Total serum levels of apolipoprotein C-II (*P* = 0.015) and apoC-III (*P* = 0.12) increased with apoC-I enrichment of VLDL particles. Neither other cardiovascular risk factors such as age, gender, smoking, BMI, blood pressure, serum lipids and apolipoproteins, hypertension and diabetes mellitus, nor cardiovascular diseases and drugs were associated with tertiles of apoC-I per VLDL particle. The distribution of apoE genotypes in our study population was 58% with the E3/3 isoform, 30% E3/4, 10% E2/3, 1% E4/4, 1% E2/4, and none had the E2/2 isoform.

The proportion of subjects with carotid plaques (*P* = 0.016) and total carotid plaque area (*P* = 0.048) increased significantly with the number of apoC-I per VLDL particle (Table 1). These trends remained significant after adjustment for age, gender, BMI, apoC-II, apoC-III, and apoE genotype (*P* = 0.011 and *P* = 0.043 for proportion of carotid plaques and plaque area, resp.). Further adjustments for cardiovascular risk factors as smoking, blood pressure, and serum lipids and apolipoproteins did not affect the relationship. 

The composition of VLDL particles with increasing enrichment of apoC-I per VLDL particle under fasting conditions are shown in Table 2. Even though no association was found between the amount of VLDL particles, assessed by the concentration of apoB within the VLDL fraction and their enrichment with apoC-I, linear regression analysis showed a marked increase in the content of triglycerides (*P* < 0.001) and modest increase in the content of cholesterol (*P* < 0.001) in VLDL particles with increasing enrichment of apoC-I. The relation between VLDL content of apoC-I and triglycerides remained significant even after adjustment for age, BMI, the content of apoC-III per VLDL particle, and apoE genotype (Table 2).

**Figure 1:**
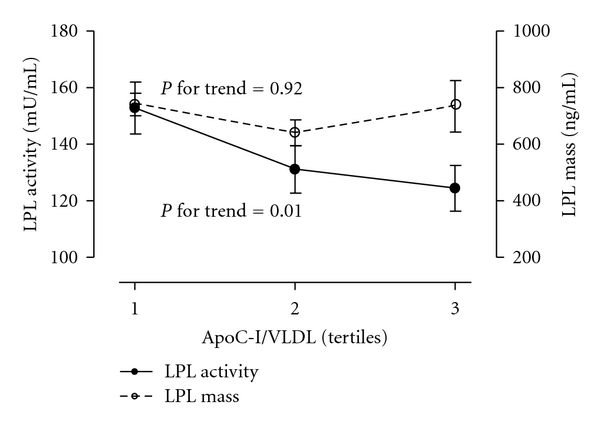
Line graph showing postheparin LPL activity and LPL mass across tertiles of the number of apoC-I per VLDL particle in the fasting state. Groups (T1–T3) were defined by their respective tertiles of the number of apoC-I per VLDL particle. Tertiles of the number of apoC-I per VLDL particle in the fasting state were as follows (mean, range): T1 (3.5, 0.5–5.0), T2 (6.2, 5.1–8.1), and T3 (12.8, 8.2–27.2). Values are means ± SEM.

We wanted to further investigate if the increased content of triglycerides in VLDL particles enriched with apoC-I was associated with less efficient hydrolysis promoted by LPL. The functional pool of LPL is anchored to heparan sulphate proteoglycans (HSPGs) at the endothelial surface, and only trace amounts are available in the circulation [29]. Unfractionated heparin has higher affinity for LPL than heparan sulphate, and heparin infusion will, therefore, displace LPL from the endothelial surface into the circulation [27]. Measurement of functional activity and mass of LPL in postheparin plasma is therefore assumed to reflect the function and bioavailability, respectively, of LPL at the endothelial surface [27]. Simple correlation analysis revealed an inverse correlation between the amount of apoC-I molecules per VLDL particle and postheparin LPL activity (*r* = −0.31, *P* = 0.006) which remained significant after adjustment for the amount of apoC-III per VLDL particle (*r* = −0.27, *P* = 0.024) but no significant association to LPL mass (*r* = −0.05, *P* = 0.65). Postheparin LPL activity decreased significantly across tertiles of VLDL particles enriched with apoC-I (*P* = 0.01) from 152.8 ± 47.4 mU/mL in T1 to 122.6 ± 40.7 mU/mL in T3 under fasting conditions, whereas LPL mass was unaffected by the apoC-I enrichment of VLDL particles (Figure 1). The relation remained significant even after adjustment for age, BMI, the content of apoC-III per VLDL, and apoE genotype (*P* = 0.013). 

**Figure 2:**
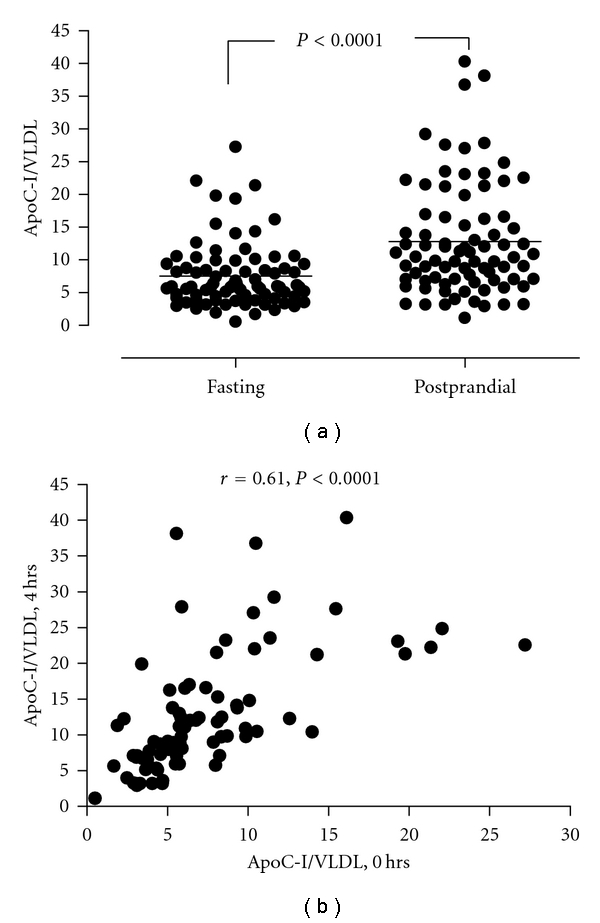
Dot plots showing the number of apoC-I per VLDL particle for each participant under fasting and postprandial conditions (a), and the relation between the number of apoC-I per VLDL particle under fasting and postprandial conditions (b).

The number of apoC-I per VLDL particle increased from 7.5 ± 4.9 under fasting conditions to 12.8 ± 6.8 under postprandial conditions (4 hrs after intake of a standard high-fat meal) (*P* < 0.0001), and simple correlation analysis revealed strong associations between the two variables (*r* = 0.61, *P* < 0.0001) (Figure 2). As seen under fasting conditions, linear regression analysis showed a marked increase in the content of triglycerides (*P* < 0.001) and modest increase in the content of cholesterol (*P* < 0.001) in VLDL particles with increasing enrichment of apoC-I in the postprandial state (Table 2). The relation between the VLDL content of apoC-I and triglycerides in the postprandial state remained significant even after adjustment for age, BMI, the content of apoC-III per VLDL particle, and apoE genotype (Table 2). Even though the number of VLDL particles did not increase in the postprandial state, their content of triglycerides and cholesterol increased from fasting to postprandial conditions across tertiles of apoC-I enrichment (Table 2).

**Figure 3:**
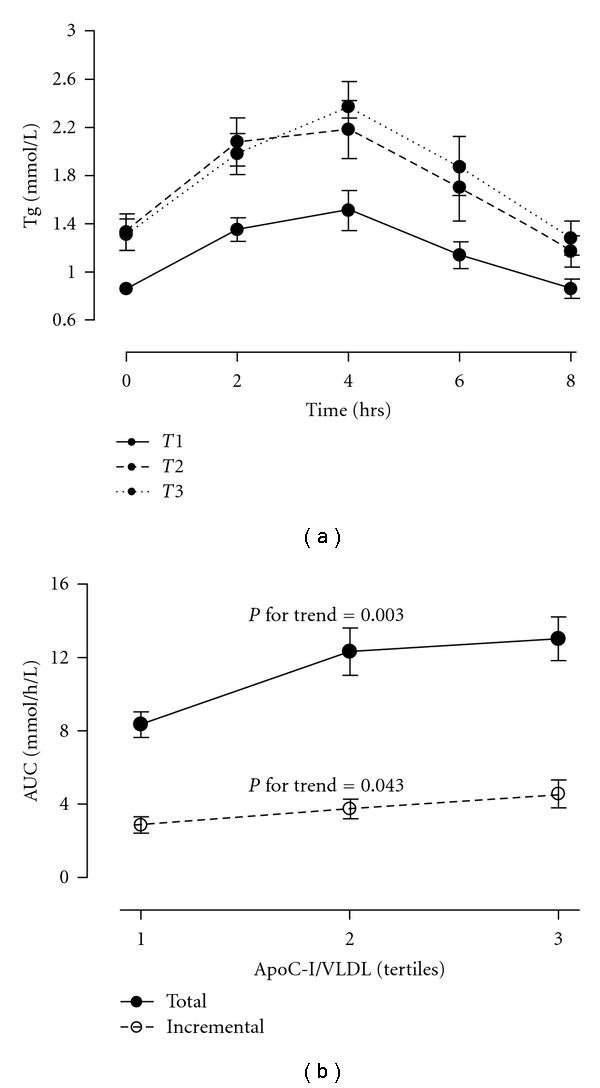
Line graphs showing serum concentrations of triglycerides (Tg) over time in groups after intake of a standard high-fat meal (a) and changes in area under the curve (AUC) for serum triglycerides in the postprandial state (0–8 hrs) across tertiles of the number of apoC-I per VLDL particle in the postprandial state (b). Groups (T1–T3) were defined by their respective tertiles of the number of apoC-I per VLDL particle in the postprandial state. Tertiles of the number of apoC-I per VLDL particle in the postprandial state were as follows (mean, range): T1 (5.3, 1.1–7.9), T2 (10.5, 8.1–13.0), and T3 (22.6, 13.7–40.3). Values are means ± SEM. Linear trend analyses were adjusted for age, BMI, the content of apoC-III per VLDL particle, and *APOE* genotyping.

To investigate if enrichment of VLDL particles with apoC-I affected postprandial lipemia, serum triglycerides were measured before and every second hour for 8 hrs after a fat tolerance test and correlated to the number of apoC-I per VLDL particle isolated in the postprandial state (4 hrs after ingestion of the meal). The amount of apoC-I molecules per VLDL was correlated with the total (*r* = 0.41, *P* < 0.0001) and incremental (*r* = 0.35, *P* < 0.001) area under the curve (AUC) of postprandial triglycerides, and the associations remained significant even after adjustment for age, BMI, the apoC-III molecules per VLDL, and apoE genotype (data not shown). The total AUC for serum triglycerides increased significantly with apoC-I enrichment of VLDL particles (*P* for trend = 0.003 in analyses adjusted for age, BMI, the content of apoC-III molecules per VLDL, and apoE genotype) from 8.4 ± 3.6 mmol/L∗h in T1 to 13.0 ± 6.1 mmol/L∗h in T3 (Figure 3). Similarly, the incremental AUC for serum triglycerides increased significantly with the number of apoC-I per VLDL particles (*P* for trend =0.043 in analyses adjusted for age, BMI, the content of apoC-III molecules per VLDL, and apoE genotype) from 2.9 ± 1.8 mmol/L∗h in T1 to 4.6 ± 2.9 mmol/L∗h in T3 (Figure 3). Postheparin LPL activity was inversely correlated with the total (*r* = −0.34, *P* = 0.002) and incremental (*r* = −0.28, *P* = 0.01) area under the postprandial triglyceride curve.

## 4. Discussion

Studies on humans [6, 30] and mice [3, 31] have shown that increased expression of apoC-I promoted combined hyperlipidemia with the most pronounced enhancing effect on plasma triglycerides. Subsequent mechanistic studies have shown that the impact of apoC-I on lipid metabolism was mainly confined to VLDL metabolism [8]. Thus, it is pertinent to focus on the impact of phenotypic expression of apoC-I in VLDLs on fasting triglycerides, postprandial lipemia, and LPL activity in humans. We found that fasting triglycerides increased linearly with apoC-I enrichment of VLDLs independent of known regulators of triglycerides such as apolipoproteins C-II, C-III, and E, apoE genotype, and traditional cardiovascular risk factors, mainly due to elevated content of triglycerides per VLDL particle. ApoC-I enrichment of VLDLs was inversely associated with plasma LPL activity, but not with LPL mass. Furthermore, the amount of apoC-I per VLDL particle isolated in the postprandial phase was associated with the magnitude of the postprandial lipemia assessed by total and incremental area under the triglyceride curve during the postprandial phase. The impact of apoC-I enrichment of VLDLs on triglyceride metabolism is probably not reflecting a shift in size distribution of VLDLs towards larger particles since previous studies have shown that VLDL particles of different sizes (Sf 20–60 and Sf 60–400) had similar amounts of apoC-I per VLDL particle [21]. Our findings provide evidence that normal variations in the content of apoC-I per VLDL particle had a substantial impact on the triglyceride metabolism, at least partly mediated by inhibition of the LPL activity.

In agreement with studies in humans [6] and mice [8, 31] with overexpression of the APOC1 gene, we found a linear increase in serum triglycerides associated with apoC-I enrichment of VLDLs. The apoC-I encoding gene (*APOC1*) is part of the *APOE/APOC1/APOC2* gene cluster [32], and apoC-I has also been shown to mediate some other lipid modifying effect by interfering with the apoE-mediated uptake of VLDLs [5, 9]. Thus, it was particularly important to investigate if the effect was independent of other important modulators of triglycerides such as apolipoproteins C-II and C-III [33, 34] and apoE genotype [35, 36]. The linear relation between apoC-I enrichment of VLDLs and fasting triglycerides remained significant after adjustment for apoC-II and apoC-III, apoE genotype, and even after further adjustments for traditional cardiovascular risk factors. This indicates that the enrichment is specific for apoC-I.

The enhanced levels of fasting triglycerides with increasing apoC-I content in VLDLs was associated with enrichment of VLDL particles by triglycerides, also reflecting increased particle size but no increased number of VLDL particles (Table 2). Previous studies in mice [8] and humans [6] with increased expression of apoC-I have also shown increased content of triglycerides in VLDLs. The mechanism beyond increased VLDL content of triglycerides may involve either increased hepatic VLDL-triglyceride production or an impaired lipolytic processing of VLDLs. Experimental studies in mice have shown that overexpression of apoC-I did not influence the hepatic VLDL-triglyceride production [8, 37]. Conversely, overexpression of apoC-I prolonged serum clearance of VLDL-like emulsion particles *in vivo* attributed to inhibition of LPL activity by apoC-I [8]. Physiological enrichment of VLDL-like TG emulsions with apoC-I inhibited the LPL activity by 33% in vitro [8], and VLDL isolated from apoC-1^+/+^ mice had a 28% decreased LPL-induced lipolysis compared to VLDL isolated from apoC1^−/−^ mice [1]. Similarly, we observed an inverse relation between apoC-I enrichment of VLDLs and LPL activity in which LPL activity decreased by 20% from the lowest to the highest tertile of apoC-I per VLDL particle. The relation between apoC-I and LPL may be due to a direct effect on the specific activity of LPL since LPL mass was unaltered across tertiles of apoC-I enrichment of VLDL particles (Figure 1). However, the molecular mechanism underlying the association between LPL activity and apoC-I remains to be elucidated.

The total and incremental area under the postprandial triglyceride curve showed a linear increase with the amount of apoC-I molecules per VLDL particle isolated under postprandial conditions. Similarly, presence of endogenous apoC-I (*APOC1^+/+^ APOE^−/−^*) in mice has shown marked elevation of postprandial triglyceride levels compared to apoC-I deficiency (*APOC1^−/−^ APOE^−/−^*) due to increased hepatic production of VLDL-triglycerides and inhibited local LPL activity *in vivo *[1]. However, no such associations were established among middle-aged men with the apoE3/E3 genotype with VLDL particles isolated 3 and 6 hrs after ingestion of the test meal [20]. The apparent discrepancy between the clinical studies may be due to differences in age, BMI, apoE genotype, which are known to influence postprandial lipemia [38, 39], and also the time for isolation of VLDL during the postprandial phase. 

In agreement with previous clinical studies [19, 20], we found a significant association between cholesterol and apoC-I enrichment within VLDL particles isolated both under fasting and postprandial conditions. In contrast, human apoC-I-expressing mice deficient of apoE did not exhibit increased cholesterol levels within the VLDL particles [8]. The molecular mechanism for the association between cholesterol and apoC-I enrichment of VLDL particles in humans and the apparent discrepancy between mice and humans are unknown. However, mice do not express CETP [40], and its function can therefore not contribute to the phenotype of APOC1 mice. Even though apoC-I appears to be a physiological relevant inhibitor of CETP, prolonged exposure in the circulation of VLDL particles enriched with apoC-I may facilitate CETP-mediated cholesterol exchange with HDL, and thereby explaining the relation between apoC-I and cholesterol enrichment in VLDLs.

The “response-to-retention” hypothesis of atherosclerosis suggested that intimal deposition is proportional to plasma levels of lipoproteins [41], and Zilversmit proposed that atherosclerosis was, at least in part, a postprandial disease due to accumulation of postprandial TRLs in the circulation and thereby exposure to the vessel wall [42]. More recent studies have shown efficient penetration and selective retention of TRLs in sites of lesion formation [43]. Elevated postprandial levels of TRLs have been associated with both coronary [44] and carotid artery atherosclerosis [45]. Similar to previous studies showing a relation between apoC-I enrichment of VLDLs and early carotid atherosclerosis [20, 21], we found a higher proportion of subjects with carotid plaques and greater plaque area among those with highest apoC-I content in VLDL. The formation of a more atherogenic composition with increased cholesterol content of VLDL particles, impaired lipolysis and subsequent delayed clearance rate of VLDLs, and elevated postprandial TRLs associated with apoC-I enrichment of VLDLs may contribute to the atherosclerotic process. Thus, we suggest that these specific modification of the triglyceride metabolism may contribute to the increased risk of atherosclerosis [18, 20, 21] and CAD [19] associated with apoC-I enrichment of VLDLs.

In conclusion, the phenotypic expression of apoC-I per VLDL particles is an important modulator of triglyceride metabolism in the fasting and postprandial states independent of apoC-III and traditional cardiovascular risk factors and may thereby represent an underlying mechanism for the association between the content of apoC-I per VLDL particle and carotid atherosclerosis.

## Figures and Tables

**Table 1:** *Characteristics of study participants.* Characteristics of subjects included in the study across tertiles of apoC-I molecules per VLDL particles (*n* = 81) under fasting conditions. Values are means ± 1 SD or percentage with number in brackets.

Variables	ApoC-I per VLDL			*P* for trend
	T1	T2	T3	
Apo-CI per VLDL	3.5 ± 1.1	6.2 ± 0.9	12.8 ± 3.1	
Men (%)	56 (15)	56 (15)	56 (15)	1.00
Age (yrs)	68.1 ± 5.5	69.3 ± 6.7	70.4 ± 6.6	0.19
Smoking (%)	19 (5)	22 (6)	22 (6)	0.68
Body mass index (kg/m^2^)	26.8 ± 3.9	26.1 ± 3.8	26.7 ± 3.9	0.93
Systolic blood pressure (mmHg)	127 ± 17	130 ± 17	132 ± 20	0.30
Diastolic blood pressure (mmHg)	74 ± 9	75 ± 11	73 ± 9	0.91
Total cholesterol (mmol/L)	6.27 ± 1.22	5.68 ± 1.63	6.26 ± 0.98	0.98
HDL cholesterol (mmol/L)	1.87 ± 0.53	1.76 ± 0.44	1.67 ± 0.46	0.13
LDL cholesterol (mmol/L)	3.89 ± 0.91	4.02 ± 1.11	3.97 ± 0.85	0.76
Triglycerides (mmol/L)	0.90 ± 0.36	1.25 ± 0.67	1.35 ± 0.77	0.01
Apolipoprotein A1 (g/L)	1.45 ± 0.27	1.42 ± 0.23	1.40 ± 0.22	0.47
Apolipoprotein B (g/L)	1.06 ± 0.20	1.07 ± 0.23	1.11 ± 0.18	0.44
Apolipoprotein C-I (mg/L)	206.7 ± 59.0	205.5 ± 65.9	215.7 ± 76.1	0.66
Apolipoprotein C-II (mg/L)	34.4 ± 10.5	40.1 ± 15.3	44.8 ± 19.1	0.015
Apolipoprotein C-III (mg/L)	107.2 ± 40.6	109.2 ± 39.7	130.8 ± 45.4	0.12
Apolipoprotein E (mg/L)	42.1 ± 12.5	40.4 ± 15.0	46.1 ± 17.4	0.34
Hypertension (%)	33 (9)	30 (8)	56 (15)	0.16
Diabetes (%)	4 (1)	19 (5)	7 (2)	0.65
Coronary artery disease (%)	19 (5)	4 (1)	30 (8)	0.29
Cerebrovascular events (%)	11 (3)	0 (0)	7 (2)	0.58
Antihypertensive drugs (%)	15 (4)	4 (1)	22 (6)	0.37
Platelet inhibitors (%)	15 (4)	11 (3)	19 (5)	0.62
Omega-3 FA supplementation (%)	52 (14)	44 (12)	56 (15)	0.79
Carotid plaques (%)	30 (8)	63 (17)	63 (17)	0.016
Plaque area (mm^2^)	4.0 ± 2.4	4.4 ± 2.7	8.8 ± 4.3	0.048

**Table 2:** *Composition of VLDL particles.* Composition of VLDL particles across tertiles of the number of apoC-I per VLDL particle isolated under fasting conditions and 4 hrs after intake of a standard high-fat meal (Postprandial conditions) (*n* = 81). Values are means ± 1 SD.

Fasting conditions (0 hrs)	ApoC-I per VLDL			*P* for trend
	T1	T2	T3	
Apo-CI per VLDL	3.5 ± 1.1	6.2 ± 0.9	12.8 ± 3.1	
ApoB (mg/L)	43.7 ± 19.2	47.3 ± 25.8	43.6 ± 23.9	0.98
Triglycerides (*μ*mol/mg apoB in VLDL)				
Crude analysis	6.8 ± 2.7	11.9 ± 5.2	15.7 ± 8.1	<0.001
Adjusted model*	6.9 ± 2.7	11.5 ± 4.7	16.3 ± 7.9	<0.001
Total cholesterol (*μ*mol/mg apoB in VLDL)				
Crude	4.9 ± 0.8	5.8 ± 1.3	6.8 ± 2.4	<0.001
Ajusted model*	4.8 ± 0.8	5.7 ± 1.3	7.0 ± 2.3	<0.001

Postprandial conditions (4 hrs)	ApoC-I per VLDL			*P* for trend
	T1	T2	T3	

Apo-CI per VLDL	5.3 ± 1.8	10.5 ± 1.5	22.6 ± 5.3	
ApoB (mg/L)	45.9 ± 23.7	52.0 ± 28.5	44.3 ± 20.2	0.81
Triglycerides (*μ*mol/mg apoB in VLDL)				
Crude analysis	13.6 ± 7.9	20.7 ± 9.8	28.1 ± 13.0	<0.001
Adjusted model*	13.1 ± 9.8	19.8 ± 8.0	29.2 ± 14.3	0.007
Total cholesterol (*μ*mol/mg apoB in VLDL)				
Crude analysis	5.5 ± 1.5	7.3 ± 2.1	7.6 ± 2.4	<0.001
Ajusted model*	5.4 ± 1.5	7.4 ± 2.2	7.7 ± 2.4	0.001

*model adjusted for age, body mass index, apoE genotype and the number of apoC-III per VLDL particle.
